# Roboterassistierte viszeralchirurgische Eingriffe in Deutschland: Eine Analyse zum aktuellen Stand sowie zu Trends der letzten 5 Jahre anhand von StuDoQ|Robotik-Registerdaten

**DOI:** 10.1007/s00104-023-01940-8

**Published:** 2023-07-27

**Authors:** Maximilian Brunner, Amr ElGendy, Axel Denz, Georg Weber, Robert Grützmann, Christian Krautz

**Affiliations:** Klink für Allgemein- und Viszeralchirurgie, Universitätsklinikum der Friedrich-Alexander-Universität Erlangen, Krankenhausstraße 12, 91054 Erlangen, Deutschland

**Keywords:** Robotische kolorektale Chirurgie, Robotische hepatopankreatobiliäre Chirurgie, Robotische Chirurgie des oberen Gastrointestinaltrakts, StuDoQ-Register, Krankenhausabrechnungsdaten, Robotic colorectal surgery, Robotic hepato-pancreato-biliary surgery, Robotic surgery of the upper gastrointestinal tract, StuDoQ register, Hospital billing data

## Abstract

Roboterassistenzsysteme werden in der Viszeralchirurgie seit einigen Jahren zunehmend häufiger eingesetzt. Entsprechend ist auch die Zahl der in Deutschland installierten Systeme rapide gestiegen. Wurden 2018 rund 100 Roboterassistenzsysteme in deutschen Kliniken genutzt, waren es 2022 bereits mehr als 200. Ziel dieser Arbeit war es, den aktuellen Entwicklungsstand und Trends der viszeralchirurgischen Roboterchirurgie in Deutschland darzustellen. Hierzu wurden Daten des StuDoQ|Robotik-Registers analysiert. Des Weiteren erfolgte eine deskriptive Analyse konkomitierender DRG-Daten über das Bundesstatistikamt (Destatis), um die Repräsentativität der StuDoQ|Robotik-Registerdaten besser abschätzen zu können. In beiden Datensätzen nahm die jährliche Zahl an roboterassistierten viszeralchirurgischen Eingriffen in Deutschland stetig zu. Im Vergleich zur DRG-Statistik waren im StuDoQ|Robotik-Register je nach Eingriffsart nur 3,7 % bis maximal 36,7 % aller durchgeführten roboterassistierten Eingriffe dokumentiert. Kolorektale Resektionen waren die häufigsten roboterassistierten Eingriffe (StuDoQ: 32,5 % und 36,7 % vs. DRG-Statistik: 24,2 % und 29,7 %) und wiesen beispielsweise niedrige Mortalitätsraten (StuDoQ: 1 % und 1 % vs. DRG-Statistik: 2,3 % und 1,3 %) auf. Aufgrund der niedrigen Erfassungsquoten roboterassistierter Ösophagus‑, Magen‑, Pankreas- und Lebereingriffe konnten für diese Bereiche keine validen Aussagen aus den StuDoQ-Daten abgeleitet werden. Mit den aktuellen Erfassungsquoten ist die Aussagekraft des StuDoQ|Robotik-Registers für einige Eingriffsarten erheblich einschränkt. In Zukunft sollten daher Wege bzw. Maßnahmen eruiert werden, die zu einer deutlichen Erhöhung der Erfassungsquoten führen.

## Abstract (en)

Robot-assisted systems have been increasingly used in general surgery for several years. Accordingly, the number of systems installed in Germany has also rapidly increased. While around 100 robot-assisted systems were used in German hospitals in 2018, this figure had already risen to more than 200 by 2022. The aim of this article is to present the current state of development and trends in robotic surgery in Germany. For this purpose, data from the StuDoQ|Robotics register were analyzed. Furthermore, a descriptive analysis of concomitant diagnosis-related groups (DRG) data was carried out via the Federal Statistical Office (Destatis), for a better assessment of the representativeness of the StuDoQ|Robotics register data. In both data sets, the annual number of robot-assisted visceral surgery procedures in Germany steadily increased. Compared to the DRG data, only 3.7% up to a maximum of 36.7% of all robot-assisted procedures performed were documented in the StuDoQ|Robotics register, depending on the type of procedure. Colorectal resections were the most frequent robot-assisted procedures (StuDoQ: 32.5% and 36.7% vs. DRG data: 24.2% and 29.7%) and had, for example, low mortality rates (StuDoQ: 1% and 1% vs. DRG data: 2.3% and 1.3%). Due to the low coverage rates of robot-assisted esophageal, gastric, pancreatic and liver interventions, no valid statements could be derived from the StuDoQ data for these areas. With the current coverage rates, the informative value of the StuDoQ|Robotics register is considerably limited for some types of intervention. In the future, measures should therefore be explored that lead to a significant increase in the coverage rates.

## Einleitung

Der Einsatz von Robotersystemen in der Chirurgie begann in den 1980er-Jahren. Vorreiter der roboterassistierten Chirurgie wurden insbesondere in der Neurochirurgie, der Urologie sowie der Gynäkologie genutzt. Seither nahm die roboterassistierte Chirurgie Einzug in viele Bereiche der Chirurgie und hat heutzutage auch einen Stellenwert in der Viszeralchirurgie [16].

Die roboterassistierte Chirurgie stellt eine konsequente Weiterentwicklung der konventionellen minimal-invasiven Chirurgie dar. Neben den bekannten Vorteilen der Laparoskopie bietet die Anwendung des Roboters zusätzliche Vorzüge (zusätzliche Freiheitsgrade, stabile 3‑D-Sicht, bessere Ergonomie), die auch unter Chirurginnen und Chirurgen zunehmend geschätzt werden. Gleichzeitig wächst die Evidenzgrundlage, die im Vergleich zur konventionellen Laparoskopie äquivalente Ergebnisse oder sogar Vorteile für roboterassistierte Eingriffe zeigt, stetig an [5, 6, 14, 21, 30]. Zuletzt wirkt auch die patientenseitige Nachfrage wahrscheinlich als Treiber für einen vermehrten Einsatz roboterassistierter Techniken. Der in diesem Zusammenhang befürchtete Wettbewerbsnachteil ist für Kliniken oft ein Grund, Roboterassistenzsysteme anzuschaffen [1]. Die mit der Roboterchirurgie verbundene Kostensteigerung stellt nach wie vor den wichtigsten negativen Faktor einer noch schnelleren Verbreitung dar [3, 12].

Aktuell werden zunehmend mehr roboterassistierte Eingriffe in der Viszeralchirurgie sowohl in Deutschland als auch weltweit durchgeführt [16, 22]. Nicht nur die Zahl der Eingriffe, sondern auch die Anwendungsbreite hat sich in den letzten Jahren vergrößert. Heutzutage sind roboterassistierte Operationstechniken für das komplette Spektrum der Viszeralchirurgie, von komplexen Leber- und Pankreaseingriffen bis hin zu Beckenexenterationen, beschrieben [13, 30].

Die folgende Arbeit beschreibt die Entwicklung der roboterassistierten Viszeralchirurgie der letzten 5 Jahre in Deutschland anhand von StuDoQ-Registerdaten und der Fallpauschalenbezogenen Krankenhausstatistik (DRG-Statistik).

## Methodik

Für diese retrospektive Auswertung wurden alle dokumentierten Eingriffe des StuDoQ|Robotik-Registers der Jahre 2017 bis 2021, welche bis zum 10.02.2022 in dieses eingegeben wurden, analysiert. Hierzu wurden Daten zur Demographie der Patienten (Alter, Geschlecht, BMI, ASA), zur Eingriffsart und zum Eingriffsjahr sowie zu perioperativen Ergebnissen (intraoperative Komplikationsrate, Konversionsrate, Krankenhausmorbidität, Krankenhaus-Major-Morbidität, 30-Tage-Mortalität, postoperative Verweildauer, Reoperationsrate, Wundheilungsstörungsrate, Wiederaufnahmerate) aus der StuDoQ|Robotik-Datenbank extrahiert und ausgewertet. Der Parameter Krankenhaus-Major-Morbidität wurde dabei definiert als jegliche Komplikation während des stationären Aufenthalts, welche gemäß der Clavien-Dindo-Klassifikation als ≥ Grad III zu werten ist [10].

Für eine vergleichende Einschätzung der Repräsentativität der Registerdaten erfolgte eine Abfrage der Fallpauschalenbezogenen Krankenhausstatistik (DRG-Statistik) mittels kontrollierter Datenfernanalyse über die Forschungsdatenzentren (FDZ) der amtlichen Statistik. Da zum Zeitpunkt der Analyse nur Daten bis zum Jahr 2020 zur Verfügung standen, wurde der Beobachtungszeitraum auf die Jahre von 2017 bis 2020 beschränkt. Es wurde die Anzahl der dokumentierten Fälle (Gesamtanzahl und Anteil an roboterassistierte Eingriffe) inklusive der Krankenhausmortalitätsraten bestimmt. Anhand geeigneter Prozedurenschlüssel erfolgte die Identifizierung und Stratifizierung aller Patienten mit entsprechenden viszeralchirurgischen Operationen während eines stationären Krankenhausaufenthaltes. Zusätzlich wurde der Anteil der Patienten, bei denen zusätzlich der OPS-Code für den Einsatz eines komplexen Operationsroboters codiert wurde, bestimmt. Für die Berechnung der Erfassungsquote des StuDoQ|Robotik-Registers wurden nur Daten aus dem Zeitraum 2017 bis 2020 verwendet (Tab. 2).

### Statistik

Es erfolgte eine deskriptive Datenanalyse. Die Daten wurden als Gesamtwerte sowie stratifiziert nach Jahren mittels Kreuztabellen dargestellt. Für die Trendanalysen wurden einfache lineare Regressionsmodelle unter Verwendung gewichteter kleinster Quadrate verwendet. Aus diesen Modellen abgeleitete *p*-Werte von weniger als 5 % wurden als Hinweis auf das Vorhandensein eines signifikanten linearen Trends während der Beobachtungsjahre betrachtet. Die statistische Auswertung erfolgte mittels SPSS-Statistikprogramm (Version 28.0, SPSS Inc., Chicago, IL, USA) bzw. mit SAS (Version 9.3 SAS Institute Inc., Cary, NC, USA).

## Ergebnisse

### Stand der roboterassistierten Chirurgie in Deutschland

**Figure 1:**
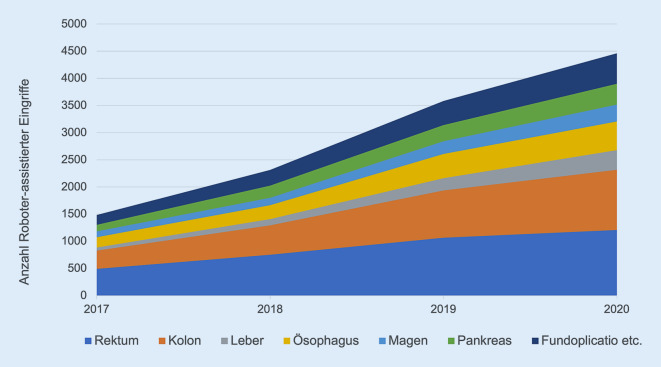

**Figure 2:**
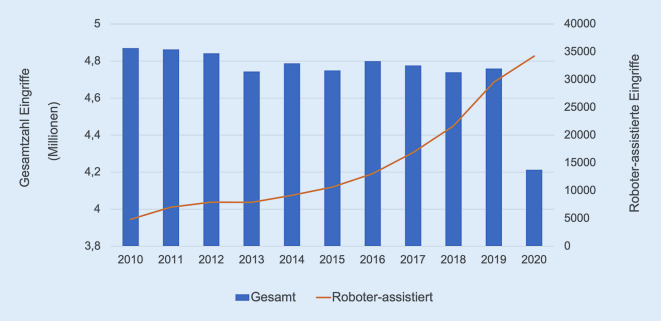

In den Jahren 2017 bis 2021 wurden insgesamt 4370 roboterassistierte Eingriffe im StuDoQ|Robotik-Register erfasst. In diesem Zeitraum haben insgesamt 59 Kliniken Daten in das Register eingegeben. Die durchschnittliche Zahl der dokumentierten Eingriffe pro Jahr und Klinik stieg von 10 im Jahr 2017 auf 30 im Jahr 2021. Analog stieg die jährliche Gesamteingriffszahl von 229 auf 1248. Die Abfrage der DRG-Statistik (FDZ-Daten) zeigt, dass in Deutschland zwischen 2017 und 2020 von insgesamt 18.488.455 Operationen 102.269 mit einem komplexen Operationsroboter durchgeführt wurden (Abb. 1). Der Anteil der roboterassistierten Chirurgie stieg in diesem Zeitraum von 0,35 % auf 0,82 %. In der Viszeralchirurgie wurden laut FDZ-Daten zwischen 2017 und 2020 von insgesamt 2.380.978 Operationen 17.995 roboterassistiert (0,76 %) durchgeführt. Die Anzahl (Abb. 2) sowie der relative Anteil viszeralchirurgischer Operationen, die mit einem komplexen Robotersystem durchgeführt wurden, stieg sowohl insgesamt als auch innerhalb jeder Eingriffsart stetig an (z. B. 2017 vs. 2020: Leber 0,33 % vs. 2,31 % bzw. Ösophagus 2,13 % vs. 5,73 %). Während im Register bis 2020 ebenfalls eine stetige Zunahme zu verzeichnen war, fand sich 2021 – a.e. bedingt durch die COVID-19-Pandemie – ein Rückgang der dokumentierten Eingriffe (2020: 1329 vs. 2021: 1248; Tab. 1). In beiden Datenquellen zählten kolorektale Resektionen zu den am häufigsten dokumentieren roboterassistierten Eingriffen (StuDoQ|Robotik Kolon: 39,0 % bzw. Rektum: 32,5 % vs. FDZ-Daten Kolon: 15,9 % bzw. Rektum: 19,5 %). Resektionen des oberen Gastrointestinaltrakts (StuDoQ|Robotik: 20,3 % vs. DRG-Daten: 8,6 %) sowie des hepatopankreatobiliären Systems (StuDoQ|Robotik 18,4 % vs. DRG-Daten: 13,6 %) hatten einen quantitativ geringeren Stellenwert.

**Table 1:** 

Organ/Eingriffsart	2017 *n* (%)	2018 *n* (%)	2019 *n* (%)	2020 *n* (%)	2021 *n* (%)	Gesamt *n* (%)
*Ösophagus*	15 (7)	23 (4)	40 (4)	47 (4)	38 (3)	163 (4)
Abdominothorakale Ösophagusresektion	13 (87)	22 (96)	33 (83)	42 (89)	35 (92)	145 (89)
Sonstiges	2 (13)	1 (4)	7 (17)	5 (11)	3 (8)	18 (11)
*Magen*	8 (3)	29 (5)	76 (8)	94 (7)	123 (10)	330 (8)
Gastrektomie	3 (10)	14 (22)	12 (10)	17 (12)	25 (14)	71 (14)
Magenteilresektion	4 (13)	5 (8)	12 (10)	24 (17)	10 (6)	55 (11)
Sonstiges	1 (3)	10 (16)	52 (45)	53 (38)	88 (50)	203 (39)
*Leber*	4 (2)	5 (1)	3 (0)	16 (1)	23 (2)	51 (1)
Lebersegmentresektion	4 (100)	5 (100)	2 (67)	9 (56)	6 (26)	26 (51)
Hemihepatektomie	0 (0)	0 (0)	0 (0)	6 (38)	14 (61)	20 (39)
Sonstiges	0 (0)	0 (0)	1 (33)	1 (6)	3 (13)	5 (10)
*Pankreas*	13 (6)	30 (5)	48 (5)	60 (5)	59 (5)	210 (5)
Pankreaskopfresektion	5 (39)	13 (43)	13 (27)	17 (28)	16 (27)	64 (31)
Pankreaslinksresektion	7 (54)	12 (40)	22 (46)	18 (30)	26 (44)	85 (41)
Pankreatektomie	1 (8)	4 (13)	5 (10)	5 (8)	5 (9)	20 (10)
Sonstiges	0 (0)	1 (3)	8 (17)	20 (33)	12 (20)	41 (20)
*Rektum*	94 (41)	243 (37)	367 (37)	396 (30)	362 (29)	1425 (33)
Rektumresektion	83 (77)	171 (70)	300 (78)	319 (79)	290 (76)	1163 (76)
Rektumextirpation	10 (9)	28 (12)	60 (16)	70 (17)	66 (17)	234 (15)
Sonstiges	1 (1)	7 (3)	7 (2)	7 (2)	6 (2)	28 (2)
*Kolon*	49 (21)	166 (30)	329 (33)	572 (43)	497 (40)	1613 (37)
Hemikolektomie rechts (inkl. erweiterte)	8 (16)	32 (19)	138 (42)	252 (44)	223 (45)	653 (41)
Hemikolektomie links (inkl. erweiterte)	5 (10)	8 (5)	18 (6)	47 (8)	40 (8)	118 (7)
Sigmaresektionen	32 (65)	106 (64)	142 (43)	226 (40)	212 (43)	718 (45)
Sonstiges	4 (8)	20 (12)	31 (9)	47 (8)	22 (4)	124 (8)
*Sonstiges*						
Fundoplicatio	23 (10)	34 (6)	39 (4)	45 (3)	54 (4)	195 (4)
Adipositas	0 (0)	0 (0)	14 (1)	13 (1)	22 (2)	49 (1)
Gallenblase	2 (1)	19 (3)	40 (4)	29 (2)	7 (1)	97 (2)
Rektopexie	14 (6)	37 (7)	18 (2)	9 (1)	19 (2)	97 (2)
Sonstiges^a^	7 (3)	14 (2)	27 (3)	48 (4)	44 (4)	140 (3)
*Gesamt*	229	563	1001	1329	1248	4370

^a^Bauchwand, Dünndarm, Milz, Nebenniere, Peritoneum, Sonstiges

Der Vergleich zwischen dem StuDoQ|Robotik-Register und der DRG-Statistik offenbarte eine deutliche Diskrepanz zwischen durchgeführten und im Register dokumentierten Eingriffen. Die Erfassungsquote des StuDoQ|Robotik-Registers lag im Durchschnitt somit bei 17,3 %. Stratifiziert nach Eingriffsart wurden im Register nur 3,7 % bis maximal 36,7 % der durchgeführten roboterassistierten Organresektionen dokumentiert (Tab. 2).

**Table 2:** 

	StuDoQ|Robotik	FDZ-Daten	Erfassungsquote StuDoQ|Robotik (%)
Ösophagusresektion	125	1333	9,4
Magenresektion	91	633	14,4
Leberresektion	28	755	3,7
Pankreasresektion	151	1016	14,9
Rektumresektion	1141	3502	32,5
Kolonresektion	1116	3042	36,7

### Patientencharakteristika und perioperative Ergebnisse bei roboterassistierten Eingriffen des oberen Gastrointestinaltrakts

Die Patientencharakteristika und perioperativen Ergebnisse bei roboterassistierten Eingriffen des oberen Gastrointestinaltrakts aus dem StuDoQ|Robotik-Register sind in Tab. 3 aufgeführt. Die Krankenhausmorbidität bzw. 30-Tage-Mortalität für roboterassistierte resezierende Ösophaguseingriffe (*n* = 145) lag bei 61 % bzw. 4 %, für roboterassistierte resezierende Mageneingriffe (*n* = 126) bei 37 % bzw. 4 %. Über die Jahre 2017 bis 2021 bestand eine tendenzielle Zunahme der Morbidität und Major-Morbidität bei roboterassistierten Ösophagusresektionen sowie ein Trend zur Abnahme der Reoperationsrate und Major-Morbidität bei roboterassistierten Magenresektionen. In der bundesweiten DRG-Statistik fand sich eine deutlich höhere Zahl an roboterassistierten Ösophagus- und Magenresektionen (Tab. 6). Die Mortalitätsraten waren zwischen StuDoQ- und FDZ-Daten (Ösophagus: 4 % vs. 4,5 %; Magen: 4 % vs. 5,4 %) vergleichbar.

**Table 3:** 

	Ösophagusresektion *n* = 145		Magenresektion *n* = 126		Fundoplicatio *n* = 195	
*Patientencharakteristika*						
Alter (Jahre)^a^	65 (15)	(=)	70 (19)	(=)	65 (19)	(=)
Geschlecht W/M (%)	21/79	(=)	48/52	(=)	62/38	(+)
BMI (kg/m^2^)^a^	24,8 (5,7)	(=)	26,2 (6,1)	(=)	28,3 (6,1)	(–)
Anteil ASA III/IV (%)	63	(=)	44	(=)	20	(=)
Anteil onkologische Eingriffe (%)	92	(=)	83	(=)	–	–
*Perioperative Ergebnisse*						
Intraoperative Komplikationen (%)	2	(+)	1	(=)	1	(=)
Konversion (%)	6	(=)	1	(=)	0	(=)
Krankenhausmorbidität (%)	61	(+)	37	(=)	14	(=)
Krankenhaus-Major-Morbidität (%)	31	(+)	18	(–)	5	(=)
30-Tage-Mortalität (%)	4	(=)	4	(=)	2	(=)
Krankenhausverweildauer (Tage)^a^	16 (12)	(=)	10 (6)	(–)	4 (3)	(=)
Reoperationsrate (%)	7	(=)	11	(–)	3	(=)
Wundheilungsstörungen (%)	4	(=)	3	(=)	1	(=)
Stationäre Wiederaufnahme (%)	4	(=)	3	(=)	0	(=)

*BMI* body mass index, *(+)* positiver, *(=)* gleichbleibender, *(–)* negativer jährlicher Trend (*p* < 0,05)

^a^Median (Interquartilbereich)

### Patientencharakteristika und perioperative Ergebnisse bei roboterassistierten hepatopankreatobiliären (HPB‑)Eingriffen

Die Patientencharakteristika und perioperativen Ergebnisse für roboterassistierte HPB-Eingriffe im im StuDoQ Register sind in Tab. 4 dargestellt. Die Krankenhausmorbidität und 30-Tage-Mortalität für roboterassistierte resezierende Pankreaseingriffe (*n* = 169) lagen bei 58 % bzw. 1 %, für roboterassistierte resezierende Lebereingriffe (*n* = 46) bei 22 % bzw. 4 %. Einziger Trend war eine Abnahme der Rate an Wundheilungsstörungen im Rahmen roboterassistierter Pankreaskopfresektionen. Im Vergleich zum StuDoQ|Robotik-Register konnten in den FDZ-Daten wesentlich mehr Eingriffe identifiziert werden (Tab. 6). Die Mortalitätsrate nach roboterassistierten Pankreasresektionen war im StuDoQ|Robotik-Register deutlich niedriger als in den FDZ-Daten.

**Table 4:** 

Operationsart	Pankreaskopfresektion *n* = 64		Pankreaslinksresektion *n* = 85		Leberresektion *n* = 46	
*Patientencharakteristika*						
Alter (Jahre)^a^	72 (12)	(=)	66 (19)	(=)	60 (17)	(=)
Geschlecht W/M (%)	41/59	(=)	56/44	(=)	50/50	(=)
BMI (kg/m^2^)^a^	25,6 (5,3)	(=)	26,2 (7,8)	(=)	26,1 (8,2)	(=)
Anteil ASA III/IV (%)	51	(=)	35	(=)	37	(=)
Anteil onkologische Eingriffe (%)	75	(=)	41	(=)	87	(=)
*Perioperative Ergebnisse*						
Intraoperative Komplikationen (%)	6	(=)	2	(=)	0	(=)
Konversion (%)	28	(=)	7	(=)	0	(=)
Krankenhausmorbidität (%)	72	(=)	48	(=)	22	(=)
Krankenhaus-Major-Morbidität (%)	33	(=)	15	(=)	9	(=)
30-Tage-Mortalität (%)	0	(=)	2	(=)	4	(=)
Krankenhausverweildauer (Tage)^a^	18 (17)	(=)	10 (6)	(=)	7 (3)	(=)
Reoperationsrate (%)	14	(=)	7	(=)	2	(=)
Wundheilungsstörungen (%)	6	(–)	2	(=)	0	(=)
Stationäre Wiederaufnahme (%)	6	(=)	8	(=)	0	(=)

*BMI* Body-Mass-Index, *(+)* positiver, *(=)* gleichbleibender, *(–)* negativer jährlicher Trend (*p* < 0,05)

^a^Median (Interquartilbereich)

### Patientencharakteristika und perioperative Ergebnisse der roboterassistierten kolorektalen Chirurgie

Die Patientencharakteristika und perioperativen Ergebnisse der roboterassistierten kolorektalen Eingriffe aus dem StuDoQ|Robotik-Register sind in Tab. 5 dargestellt. Die Krankenhausmorbidität bzw. 30-Tage-Mortalität für resezierende Rektumeingriffe (*n* = 1397) lag bei 34 % bzw. 1 %, für resezierende Koloneingriffe (*n* = 1489) bei 25 % bzw. 1 %. Die Trendanalyse der perioperativen Ergebnisse über die Jahre 2017 bis 2021 zeigte mehrere positive Entwicklungen: einen Trend zu einer niedrigeren Konversionsrate sowie einer geringeren Mortalität bei Rektumresektionen, zu einer niedrigeren Konversionsrate, einer geringeren Major-Morbidität, einer kürzeren postoperativen Verweildauer, einer niedrigeren Reoperationsrate sowie einer geringeren Rate an Wundheilungsstörungen bei Rektumextirpationen, zu einer geringeren Morbidität und einer kürzeren postoperativen Verweildauer bei Rechtshemikolektomien sowie zu einer geringeren Morbidität, einer kürzeren postoperativen Verweildauer und einer geringeren Rate an Wundheilungsstörungen bei Sigmaresektionen. Für Kolon- und Rektumresektionen zeigten sich die höchsten Erfassungsquoten (32,5 % und 36,7 %) im StuDoQ|Robotik-Register. Dementsprechend konnte jeweils eine hohe Fallzahl (*n* = 1163 und *n* = 1489) identifiziert werden. Im Vergleich zur DRG-Statistik waren die Mortalitätsraten im StuDoQ|Robotik-Register geringfügig niedriger (Tab. 6).

**Table 5:** 

	Rektumresektion (*n* = 1163)		Rektumexstirpation (*n* = 234)		Hemikolektomie rechts (*n* = 653)		Hemikolektomie links (*n* = 118)		Sigmaresektion (*n* = 718)	
*Patientencharakteristika*										
Alter (Jahre)^a^	66 (17)	(+)	69 (21)	(=)	74 (16)	(=)	74 (16)	(=)	63 (19)	(+)
Geschlecht W/M (%)	36/64	(=)	36/64	(=)	48/52	(=)	41/59	(=)	48/52	(=)
BMI (kg/m^2^)^a^	25,9 (6,1)	(–)	25,7 (5,8)	(=)	25,5 (5,2)	(=)	26,4 (6,1)	(=)	26,6 (6,8)	(=)
Anteil ASA III/IV (%)	41	(=)	50	(=)	52	(+)	60	(=)	34	(+)
Anteil onkologische Eingriffe (%)	97	(=)	94	(=)	89	(+)	93	(+)	38	(+)
*Perioperative Ergebnisse*										
Intraoperative Komplikationen (%)	2	(=)	3	(=)	1	(=)	3	(=)	0	(=)
Konversion (%)	9	(–)	8	(–)	9	(=)	10	(=)	4	(=)
Krankenhausmorbidität (%)	33	(=)	38	(=)	27	(–)	31	(=)	23	(–)
Krankenhaus-Major-Morbidität (%)	16	(=)	15	(–)	7	(=)	15	(=)	9	(=)
30-Tage-Mortalität (%)	1	(–)	3	(=)	1	(=)	2	(=)	1	(=)
Krankenhausverweildauer (Tage)^a^	10 (7)	(=)	11 (8)	(–)	7 (4)	(–)	8 (5)	(=)	7 (3)	(–)
Reoperationsrate (%)	10	(=)	10	(–)	4	(=)	13	(=)	7	(=)
Wundheilungsstörungen (%)	7	(=)	15	(–)	4	(=)	7	(=)	7	(–)
Stationäre Wiederaufnahme (%)	8	(=)	10	(=)	4	(=)	5	(=)	4	(=)

*BMI* Body-Mass-Index, *(+)* positiver, (=) gleichbleibender, (–) negativer jährlicher Trend (*p* < 0,05)

^a^Median (Interquartilbereich)

**Table 6:** 

Eingriffsart		StuDoQ|Robotik (2017–2021)	FDZ-Daten (Robotik^a^) (2017–2020)	FDZ-Daten (gesamt) Literatur^b^	Übersichtsdaten (Robotik) Internationale Literatur
Ösophagusresektion	*n*	145	1333	22.700 [25]	181 [29]; 974 [2]
	Verweildauer (Tage)	16^c^	24,3	20–22^c^ [25]	9^c^ [29]; 12,1 [2]
	Morbidität (%)	61	–	52,4–57,5 [25]	48,6 [29]
	Mortalität (%)	4	4,5	6,8–11,4^f^ [25]	0,8 [29]
Magenresektion	*n*	126	663	72.528 [24]	185 [15]; 1134 [28]
	Verweildauer (Tage)	10^c^	16,4	17–20^c^ [24]	6^c^ [15]; 6,6 [28]
	Morbidität (%)	37	–	39,0–41,1 [24]	13,5^e^ [15]; 11,7 [28]
	Mortalität (%)	4	5,4	10,6–12,0^f^ [24]	0,0^e^ [15]; 0,4 [28]
Pankreasresektion	*n*	169	1016	60.858 [18]	500 [30]
	Verweildauer (Tage)	10–18^c^	20,4	20–25^c^ [18]	8^c,d^ [30]
	Morbidität (%)	58	–	–	68,8^d^ [30]
	Mortalität (%)	1	4,5	6,5–11,5^f^ [18]; 10,1 [26]	0,8^d^ [30]
Leberresektion	*n*	46	755	31.114 [17]	77 [20], 458 [19]
	Verweildauer (Tage)	7^c^	–	16–18^c^ [17]	5^c^ [20]; 4,0 [19]
	Morbidität (%)	22	–	33,3–39,7 [17]	15,0 [20]; 15,5 [19]
	Mortalität (%)	4	–	7,4–11,4^f^ [17]	0,0 [20]; 0,5 [19]
Rektumresektion	*n*	1163	3502	64.349 [9]	334 [27]
	Verweildauer (Tage)	10–11^c^	15,2	19,7–21,7 [9]	8,3 [27]
	Morbidität (%)	33	–	–	27,3^e^ [27]
	Mortalität (%)	1	1,3	2,6–5,3 [9]	0,6^e^ [27]
Kolonresektion	*n*	1489	3042	129.196 [8]	1740 [7]
	Verweildauer (Tage)	7–8	13,6	18,7–20,3 [8]	6,5 [7]
	Morbidität (%)	25	–	–	17,4 [7]
	Mortalität (%)	1	2,3	4,8–6,9 [8]	0,3 [7]

^a^FDZ-Daten (DRG-Statistik) 2017–2020 nur roboterassistierte Eingriffe

^b^Analysen der DRG-Statistik ohne Stratifizierung nach Zugangsweg

^c^Median

^d^nur Pankreaskopfresektion

^e^nur onkologische Eingriffe

^f^risikoadjustiert

## Diskussion

Mit dieser Arbeit konnte erstmalig ein Überblick zur Robotik in der Viszeralchirurgie in Deutschland gewonnen werden. Die öffentlich oft diskutierte zunehmende Implementierung roboterassistierter Verfahren in der Viszeralchirurgie wurde in unserer Analyse durch zunehmende Eingriffszahlen im StuDoQ|Robotik-Register und in den FDZ-Daten bestätigt [16]. Dennoch war der Anteil roboterassistierter Operationen an der Gesamtzahl bestimmter Eingriffsarten im Jahr 2020 immer noch sehr gering (FDZ-Daten: z. B. Kolonresektionen 2,4 % bzw. Rektumresektionen 5,8 %). Somit entspricht der mitunter wahrgenommene „Robotik-Hype“ nicht (zumindest noch nicht) der eigentlichen Versorgungsrealität.

Die vorliegende Arbeit konnte zeigen, dass die Erfassungsquoten des StuDoQ|Robotik-Registers stark schwankten und auf einem niedrigen (Ösophagus‑, Magen‑, Pankreas- und Lebereingriffe) bis mittleren (Kolon- und Rektumeingriffe) Niveau lagen. Die untersuchten StuDoQ|Robotik-Daten hatten somit keinerlei Aussagekraft hinsichtlich der versorgungsrealitätsrelevanter Fragestellungen. Aufgrund der stark limitierten Fallzahlen für Ösophagus‑, Magen‑, Pankreas- und Lebereingriffe waren auch die Trendanalysen und die Benchmarkvergleiche (FDZ-Daten oder Literatur) in diesen Bereichen nicht aussagekräftig genug. Demgegenüber ermöglichte jedoch die beachtliche Datenmenge für kolorektale Eingriffe relevante Aussagen.

Die Tatsache, dass Roboterassistenzsysteme in der Viszeralchirurgie in Deutschland mehrheitlich bei kolorektalen Eingriffen zum Einsatz kamen, entspricht der Beobachtung, dass für diesen Bereich bislang wahrscheinlich weltweit die größte Erfahrung als auch Evidenz durch randomisierte kontrollierte Studien vorhanden ist. In letzteren zeichneten sich zuletzt sogar Vorteile der roboterassistierten Rektumresektion gegenüber dem konventionell-laparoskopischen Verfahren ab, welche sich insbesondere in einer niedrigeren Konversionsrate, aber in einigen Studien auch in einer niedrigeren Morbidität und Mortalität, in einem kürzeren Krankenhausaufenthalt sowie besseren funktionellen Ergebnissen bei gleicher onkologischer Qualität und erhöhter Operationszeit abbildeten [4, 5, 11, 23].

In den StuDoQ|Robotik-Daten für kolorektale Eingriffe zeigte sich im Verlauf der letzten fünf Jahre für verschiedene perioperative Ergebnisparameter (Morbidität, Mortalität und postoperative Verweildauer) ein Trend zur Verbesserung, was auf eine zunehmende Erfahrung und Routine in der roboterassistierten kolorektalen Chirurgie in Deutschland hindeuten könnte. Im Benchmarkvergleich mit FZD-Daten (Robotik) und weltweiten Übersichtsdaten ergaben sich keine größeren Abweichungen (Tab. 6), was eine hohe Qualität der roboterassistierten kolorektalen Chirurgie in den eingebenden Kliniken vermuten lässt [7, 27]. Der Vergleich zu FZD-Daten aus der Literatur (ohne Stratifizierung nach Zugangsweg) zeigte erwartungsgemäß aufgrund des Selektionsbias niedrigere Mortalitätsraten und eine kürzere Krankenhausverweildauer bei roboterassistierten Eingriffen [8, 9, 17, 18, 24–26]. Die FZD-Daten für roboterassistierte Ösophagus‑, Magen‑, Pankreas- und Lebereingriffe ergaben höhere Mortalitätsraten und eine längere Krankenhausverweildauer im Vergleich zu weltweiten Übersichtsdaten [2, 15, 19, 20, 28–30]. Allerdings haben diese Vergleiche aufgrund der unterschiedlichen Datengrundlagen allenfalls eine sehr eingeschränkte Aussagekraft.

Die gezeigten StuDoQ|Robotik-Daten unterliegen neben der oben genannten Einschränkung aufgrund der niedrigen bis mittleren Erfassungsquoten noch weiteren Limitationen. Es könnte ein Selektionsbias hinsichtlich der Eingabe von Patientendaten in das StuDoQ|Robotik-Register bestehen. Allerdings wird von StuDoQ jährlich ein Vollständigkeitsreport gefordert, welcher einem solchen Selektionsbias entgegenwirken soll. Eine Limitation der Daten aus dem Jahre 2021 besteht zudem in der Tatsache, dass die Dokumentation der Daten des Jahres 2021 meist erst mit einer Verzögerung vorgenommen wird, weshalb es sehr wahrscheinlich ist, dass zum Zeitpunkt der Datenabfrage im Februar 2022 noch nicht alle Fälle des Jahres 2021 im StuDoQ|Robotik-Register eingetragen waren.

## Fazit

In Deutschland nahm die Anzahl roboterassistierter Eingriffe in der Viszeralchirurgie zwischen 2017 und 2020 stetig zu. Gemessen am prozentualen Anteil aller Eingriffe war die Robotik in diesem Zeitraum nicht flächendeckend implementiert. Roboterassistierte kolorektale Eingriffe spielten anteilig die größte Rolle und wurden auch im Vergleich zu weltweiten Übersichtdaten mit einer hohen Ergebnisqualität durchgeführt. Für die Bereiche Ösophagus‑, Magen‑, Pankreas- und Leberchirurgie zeigten sich im StuDoQ|Robotik-Register niedrige Erfassungsquoten, welche keine validen Aussagen zum aktuellen Entwicklungsstand oder zur Ergebnisqualität zuließen. Da die Einführung neuer Techniken immer Chancen und Risiken beinhaltet und sich hierbei eine begleitende Bewertung anhand von Registerdaten in der Vergangenheit bewährt hat, sollte von Seiten der DGAV eine deutliche Erhöhung der Erfassungsquoten angestrebt werden. Letztere könnte beispielsweise über eine Vergabe von Zertifizierungen zum „Robotische Spezialistin bzw. Robotischer Spezialist“ durch die DGAV (analog der Zertifizierung „Endovaskulärer Spezialist“ der DGG) erreicht werden, sofern solche Zertifikate u. a. mit einer verpflichtenden Teilnahme am StuDoQ|Robotik-Register verknüpft sind.

## References

[1] Ahmad A, Ahmad ZF, Carleton JD, Agarwala A (2017). Robotic surgery: current perceptions and the clinical evidence. Surg Endosc.

[2] Angeramo CA, Harriott BC, Casas MA, Schlottmann F (2021). Minimally invasive Ivor Lewis esophagectomy: Robot-assisted versus laparoscopic–thoracoscopic technique. Systematic review and meta-analysis. Surgery.

[3] Brunner M, Matzel K, Aladashvili A et al (2019) Implementierung eines Roboterprogramms in der Viszeralchirurgie – Erfahrungen eines deutschen Zentrums. Zentralblatt Für Chir – Z Für Allg Visz Thorax-Gefäßchirurgie 144:224–234. 10.1055/a-0600-9868 10.1055/a-0600-9868 29775978

[4] Corrigan N, Marshall H, Croft J (2018). Exploring and adjusting for potential learning effects in ROLARR: a randomised controlled trial comparing robotic-assisted vs. standard laparoscopic surgery for rectal cancer resection. Trials.

[5] Crippa J, Grass F, Dozois EJ (2021). Robotic Surgery for Rectal Cancer Provides Advantageous Outcomes Over Laparoscopic Approach: Results From a Large Retrospective Cohort. Ann.

[6] Croner RS, Perrakis A, Hohenberger W, Brunner M (2016). Robotic liver surgery for minor hepatic resections: a comparison with laparoscopic and open standard procedures. Langenbecks Arch Surg.

[7] Cuk P, Kjær MD, Mogensen CB (2022). Short-term outcomes in robot-assisted compared to laparoscopic colon cancer resections: a systematic review and meta-analysis. Surg Endosc.

[8] Diers J, Wagner J, Baum P (2019). Nationwide in-hospital mortality following colonic cancer resection according to hospital volume in.

[9] Diers J, Wagner J, Baum P (2020). Nationwide in-hospital mortality rate following rectal resection for rectal cancer according to annual hospital volume in.

[10] Dindo D, Demartines N, Clavien P‑A (2004) Classification of Surgical Complications: A New Proposal With Evaluation in a Cohort of 6336 Patients and Results of a Survey. Ann Surg 240:205–213. 10.1097/01.sla.0000133083.54934.ae 10.1097/01.sla.0000133083.54934.ae PMC1360123 15273542

[11] Feng Q, Yuan W, Li T et al (2022) Robotic versus laparoscopic surgery for middle and low rectal cancer (REAL): short-term outcomes of a multicentre randomised controlled trial. Lancet Gastroenterol Hepatol 7:991–1004. 10.1016/S2468-1253(22)00248‑5 10.1016/S2468-1253(22)00248-5 36087608

[12] Higgins RM, Frelich MJ, Bosler ME, Gould JC (2017). Cost analysis of robotic versus laparoscopic general surgery procedures. Surg Endosc.

[13] Ishizaki T, Mazaki J, Enomoto M (2022). A new technique for robotic lateral pelvic lymph node dissection for advanced low rectal cancer with emphasis on en bloc resection and inferior vesical vessel preservation. Surg Endosc.

[14] Jayne D, Pigazzi A, Marshall H et al (2017) Effect of Robotic-Assisted vs Conventional Laparoscopic Surgery on Risk of Conversion to Open Laparotomy Among Patients Undergoing Resection for Rectal Cancer: The ROLARR Randomized Clinical Trial. JAMA 318:1569–1580. 10.1001/jama.2017.7219 10.1001/jama.2017.7219 PMC5818805 29067426

[15] Kim MS, Kim WJ, Hyung WJ (2021). Comprehensive Learning Curve of Robotic Surgery: Discovery From a Multicenter Prospective Trial of Robotic Gastrectomy. Ann.

[16] Kissler HJ, Bauschke A, Settmacher U (2016). Erste nationale Umfrage zum Operationsrobotereinsatz in der Viszeralchirurgie in Deutschland. Chir.

[17] Krautz C, Gall C, Gefeller O (2020). In-hospital mortality and failure to rescue following hepatobiliary surgery in Germany—a nationwide analysis. BMC Surg.

[18] Krautz C, Nimptsch U, Weber GF (2018). Effect of hospital volume on in-hospital morbidity and mortality following pancreatic surgery in Germany. Ann Surg.

[19] Machairas N, Papaconstantinou D, Tsilimigras DI (2019). Comparison between robotic and open liver resection: a systematic review and meta-analysis of short-term outcomes. Updat Surg.

[20] Masetti M, Fallani G, Ratti F (2022). Minimally invasive treatment of colorectal liver metastases: does robotic surgery provide any technical advantages over laparoscopy? A multicenter analysis from the IGoMILS (Italian Group of Minimally Invasive Liver Surgery) registry. Updat Surg.

[21] Muaddi H, Hafid ME, Choi WJ (2021). Clinical Outcomes of Robotic Surgery Compared to Conventional Surgical Approaches (Laparoscopic or Open): A Systematic Overview of Reviews. Ann.

[22] Muaddi H, Stukel TA, De Mestral C (2023). The evolving use of robotic surgery: a population-based analysis. Surg Endosc.

[23] Ng KT, Tsia AKV, Chong VYL (2019). Robotic Versus Conventional Laparoscopic Surgery for Colorectal Cancer: A Systematic Review and Meta-Analysis with Trial Sequential Analysis. World J Surg.

[24] Nimptsch U, Haist T, Gockel I (2019). Complex gastric surgery in Germany—is centralization beneficial? Observational study using national hospital discharge data. Langenbecks Arch Surg.

[25] Nimptsch U, Haist T, Krautz C (2018). Hospital Volume, In-Hospital Mortality, and Failure to Rescue in Esophageal Surgery. Dtsch Ärztebl Int.

[26] Nimptsch U, Krautz C, Weber GF (2016). Nationwide In-hospital Mortality Following Pancreatic Surgery in.

[27] Prete FP, Pezzolla A, Prete F (2018). Robotic.

[28] Wang Y, Zhao X, Song Y et al (2017) A systematic review and meta-analysis of robot-assisted versus laparoscopically assisted gastrectomy for gastric cancer. Medicine (Baltimore) 96:e8797. 10.1097/MD.0000000000008797 10.1097/MD.0000000000008797 PMC5728759 29310358

[29] Yang Y, Li B, Yi J (2022). Robot-assisted.

[30] Zureikat AH, Beane JD, Zenati MS (2021). 500 Minimally Invasive Robotic Pancreatoduodenectomies: One Decade of Optimizing.

